# Pioneering sustainable treatment delivery in childhood leukemia through synchronous telemedicine—A pilot study

**DOI:** 10.1002/ijc.35253

**Published:** 2024-11-09

**Authors:** Andreas Meryk, Christina Salvador, Gabriele Kropshofer, Benjamin Hetzer, Gerhard Rumpold, Alexandra Haid, Verena Schneeberger‐Carta, Bernhard Holzner, Roman Crazzolara

**Affiliations:** ^1^ Department of Pediatrics Medical University of Innsbruck Innsbruck Austria; ^2^ Department of Psychiatry, Psychotherapy and Psychosomatics University Hospital of Medical Psychology, Medical University of Innsbruck Innsbruck Austria; ^3^ Department of Psychiatry, Psychotherapy and Psychosomatics University Hospital of Psychiatry II, Medical University of Innsbruck Innsbruck Austria

**Keywords:** acute leukemia, integrated health care network, telemedicine, travel burden

## Abstract

Cancer care places a heavy economic burden on families and health systems, driven by high treatment costs, lengthy hospital stays, and the necessity for extensive travel to specialized facilities. To address this challenge, an integrated health care network (IHCN) was implemented for maintenance treatment in acute leukemia. The IHCN encompassed outpatient services provided by local physicians and synchronous telemedicine consultation with pediatric oncologists. This study included twenty‐two pediatric patients (eleven [50.0%] females; twenty [90.9%] with B‐ALL and two [9.1%] with AML). The IHCN was offered to all rural patients (*n* = 17) with a one‐way driving distance more than 30 km, while urban patients (*n* = 5) received regular cancer care. Throughout the study, rural patients had a total of 510 routine clinical visits, with 367 (72%) conducted through the IHCN. Physical examinations revealed similar frequency of new abnormal findings for urban and rural patients (22.4% vs. 17.8%; *p* = .31). Laboratory tests indicated no significant difference in the frequency of abnormal values for various parameters between both groups. Similarly, there was no discrepancy of drug modifications or interruption in maintenance therapy between the two settings (*p* = .85). Moreover, patients' health‐related quality of life remained within the normative range, and user satisfaction with the IHCN was notably high. The implementation of the IHCN resulted in savings of 70,158 km, 950 h of travel, and 12,277 kg CO_2_ emissions. This pilot study underscores the efficacy of a telemedicine‐based IHCN, ensuring safety, quality of care, cost reduction, and satisfaction for both families and health care providers in pediatric leukemia management.

## Graphical abstract

What's new?

Cancer care requires frequent traveling to centralized health care services, placing an additional burden on patients from rural areas. This prospective study aimed to implement a telemedicine‐based integrated health care network for maintenance treatment in acute childhood leukemia. The network provided outpatient services imparted by local physicians and synchronous telemedicine consultations with pediatric oncologists. The findings demonstrate that a telemedicine‐based integrated health care network can be implemented in the routine clinical care of pediatric leukemia patients and may ensure quality of care, reduced travel costs, safety, and satisfaction from both families and clinicians.

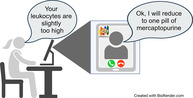

## 1 INTRODUCTION

Significant progress has been made in treating childhood cancer, with survival rates now exceeding 90%.^1^, ^2^ Despite this success, one significant hurdle is the burden of travel, particularly for those living far from specialized hospitals, impacting them physically, emotionally, and financially. While much attention has been given to the United States, where health care operates on a user‐pays system,^3^, ^4^ families in countries with publicly‐funded health care systems also encounter financial hurdles related to homecare, hospital fees, travel and accommodation, and income losses.^5^ Addressing these obstacles is essential to ensure equitable access to high‐quality care for all pediatric cancer patients and to optimize treatment outcomes. Digital health care technologies offer promising solutions for reducing the necessity for in‐person visits while enhancing the overall patient experience in terms of quality and satisfaction.^6^, ^7^

The COVID‐19 pandemic gave a significant global boost to innovative digital approaches in health care. Many highly recommended telemedicine services were introduced into clinical practice for adult cancer patients across various care settings, such as cancer care, palliative care and, rehabilitation.^8^, ^9^, ^10^ Some of these services reported a significant increase in virtual care visits, accounting for up to 70% of all appointments. They also led to direct and indirect cost savings, reduced carbon emissions, and maintained quality performance.^11^, ^12^, ^13^, ^14^ Despite its clear advantages, there are concerns about the quality of patient care, liability and regulatory issues, credentialing and licensing processes, and reimbursement.^15^ Additional barriers for both patients and health care providers include limited financial resources, staff shortages, and challenges with technology use, particularly among older adults.^16^, ^17^ Yet, there remains uncertainty about whether telemedicine services might be feasible and have such potential in children with acute leukemia, as there are no studies in this area to date.

In treating pediatric acute leukemia, the standard approach involves an initial phase of intensive chemotherapy lasting around 6–9 months, followed by maintenance therapy for up to 1.5 years. During this maintenance phase, regular clinical visits with physical examinations and blood tests are necessary to adjust drug therapy, which includes medications like 6‐mercaptopurine, methotrexate, and thioguanine. A few years ago, we developed a mobile application called ePROtect for ongoing health monitoring of childhood cancer patients^18^, ^19^, ^20^ and now aim to integrate it into a telemedicine‐based integrated health care network (IHCN). Our goal is to evaluate the feasibility of using synchronous telemedicine for routine clinical care during the maintenance therapy phase. This was assessed by the frequency of in‐person visits at the Medical University of Innsbruck (MUI) that were replaced by telemedicine. Additional factors included savings on travel time and costs, as well as patient satisfaction with the IHCN, measured using the telemedicine usability questionnaire (TUQ). Patients' safety and quality of care were also evaluated by the frequency and types of adverse events as well as the continuity of maintenance therapy. To achieve this, patients exposed to telemedicine were compared with those receiving regular cancer care without telemedicine.

## 2 METHODS

### 2.1 Participants

This single‐center cohort study was conducted from March 1, 2023, to February 29, 2024, at the MUI. All children and adolescents with acute leukemia undergoing maintenance therapy according to the following study protocols were recruited: AIEOP‐BFM ALL 2017 (V3.0_22.11.2021 and V4.0_28.04.2023; frontline therapy for acute lymphoblastic leukemia [ALL]), IntReALL SR 2010 (V2.1_09.12.2019; therapy for ALL relapse) and treatment recommendations of the AML‐BFM study group (V1.0_19.07.2019; acute myeloid leukemia [AML] therapy). In contrast to non‐BFM protocols, our study group continues with maintenance therapy in AML patients after reinduction for the duration of 1 year. Patients were followed up until completion of maintenance therapy or during ongoing therapy until February 29, 2024.

### 2.2 Study design

Patients were categorized as either urban or rural based on a one‐way driving distance threshold of 30 km, which is the geographical midpoint in the distance to the nearest hospital where pediatric care is available. Urban patients living within 30 km to the MUI received standard care according to the guidelines without any telemedicine use (regular cancer care). Rural patients with a one‐way travel distance >30 km (telemedicine‐centered cancer care) were offered the synchronous telemedicine‐based IHCN. The IHCN encompassed outpatient services provided by local physicians, ensuring access to comprehensive medical assessments including physical examination with vital sign monitoring. Additionally, patients received essential laboratory tests such as complete blood counts, C‐reactive protein (CRP) concentration assessments, and gamma‐glutamyl transferase levels. These diagnostic procedures were conducted either at the local hospital or by the general practitioner during morning hours, ensuring timely and efficient health care delivery. The results of the clinical examination were immediately sent by fax or email and added to the electronic patient chart at MUI. Video consultation with the family and the pediatric oncologists from the MUI were facilitated in the afternoon of the same day through the ePROtect app.^18^ Patients and families were trained in the use of the ePROtect software by the professional health care team.^18^, ^19^ The findings from the clinical examination were thoroughly reviewed, leading to adjustments of maintenance therapy based on blood results. Specifically, dosages of medications such as 6‐mercaptopurine, methotrexate, and thioguanine were modified accordingly. The frequency of appointments was determined to be either once‐ or biweekly, contingent upon the necessity to adjust the treatment regimen. Specific dates and times were booked through the app and the family was responsible for arranging the next appointment with the local physicians. In the event of unforeseen health deterioration, such as fever, patients and their families were instructed to promptly reach out to the pediatric oncologists at MUI for immediate assistance. In brief, pediatric oncologists from the MUI were responsible for drug monitoring and treatment decisions, while local physicians assisted in collecting necessary data and observing patients. Visits designated for administration of subcutaneous or intrathecal administration of chemotherapy, as well as appointments coinciding with other clinic departments, were excluded from the analysis.

### 2.3 Measurement tools and assessment

The proxy‐specific versions of the Pediatric Quality of Life Inventory (PedsQL) 4.0 Generic Core Scales, a multidimensional measure of general health‐related quality of life (HRQOL), and the PedsQL 3.0 Cancer Module which focuses on the dimensions of health affected by pediatric cancer and its treatment, were used.^21^ The PedsQL Generic contains 23 items forming four principal domains including physical functioning (8 items), emotional functioning (5 items), school functioning (5 items), and social functioning (5 items). The PedsQL Cancer comprises 27 items in eight subscales (level of pain (2), nausea (5), procedural anxiety (3), treatment anxiety (3), worry (3), cognitive problems (5), perceived physical appearance (3), and communication (3)). In addition, satisfaction of parents as well as physicians with IHCN was collected anonymously using the TUQ (German version provided by Salzwedel^22^). The TUQ (range, 1–7; higher score is better) consists of a 21‐item questionnaire assigned to six subscales as indicated by the respective grading “usefulness,” “ease of use and learnability,” “interface quality,” “interaction quality,” “reliability,” and “satisfaction and future use” with an average score of >5 considered as usable.^23^

### 2.4 Data collection

We collected patients' sociodemographic and basic clinical data at study inclusion. Abnormal physical findings and adverse events occurring within the study period were allocated to the International Statistical Classification of Diseases and Related Health Problems (ICD‐10) (v2019) and recorded along with the required interventions in the electronic medical chart. CRP concentrations over 0.7 mg/dL were considered as elevated, and levels of gamma‐glutamyl transferase were categorized according to the Common Terminology Criteria for Adverse Events version 5.0 (CTCAE) criteria. Overall travel savings were estimated using Google Maps as the difference between the patient's home and the MUI as compared with outpatient visits by local physicians. The kilometer rate of € 0.42 set by the Austrian tax authority was used to calculate direct transportation costs. The CO_2_ value was set at 0.175 kg/km, which corresponds to a gasoline vehicle with an average consumption of 7.4 L/100 km in European countries.

### 2.5 Outcome measurements

Our primary focus was on implementing a synchronous telemedicine‐based IHCN to substitute in‐person visits at the MUI. Secondary objectives encompassed assessing safety and quality of care by the frequency and types of adverse events as well as continuity of maintenance therapy. Additionally, savings on cost of transport were estimated using Google Maps, and overall satisfaction with IHCN was measured using the TUQ.

### 2.6 Statistical analysis

Sample characteristics were calculated as absolute numbers, percentages, median, mean, interquartile range (IQR), and standard deviation (SD). The Mann–Whitney *U* test and Chi‐Square test were used for comparisons. Differences were considered statistically significant at *p* < .05. All statistical analyses were performed using SPSS 26.0, and for data visualization, GraphPad Prism (version 8.4) was used.

## 3 RESULTS

### 3.1 Patient characteristics

Twenty‐two patients with acute leukemia undergoing maintenance therapy at MUI were screened between March 2023 and February 2024 (eleven [50.0%] females; twenty [90.9%] with B‐ALL and two [9.1%] with AML; four [18.2%] had a relapse). Based on a one‐way driving distance threshold of 30 km, patients were classified as either urban (<30 km) or rural (>30 km). Urban patients (*n* = 5) received regular cancer care, while rural patients (*n* = 17) were offered telemedicine‐based IHCN. All of them participated without any early withdrawal (100% enrollment rate). Baseline demographics and disease characteristics were similar between the two groups, except for the proportion of high‐risk patients (80.0% vs. 17.6%, urban vs. rural, respectively; *p* = .009; Table 1).

**TABLE 1:** Demographic and clinical characteristics of the study cohort.

Characteristics	Regular cancer care (urban, *N* = 5)	Telemedicine‐centered cancer care (rural, *N* = 17)	*p‐*value
Age, median (IQR), years	5.0 (3.0–5.8)	3.9 (2.6–6.8)	.820
Sex			.611
Female	3 (60.0)	8 (47.1)	
Male	2 (40.0)	9 (52.9)	
Underlying diagnosis			.334
B‐ALL	4 (80.0)	16 (94.1)	
AML	1 (20.0)	1 (5.9)	
Risk groups			.009
Standard and intermediate	1 (20.0)	14 (82.4)	
High	4 (80.0)	3 (17.6)	
Relapse	1 (20.0)	3 (17.6)	.905
Travel conditions to MUI			
Distance, median (IQR), km	25 (23–26)	218 (154–280)	<.001
Time, median (IQR), min	28 (28–42)	162 (130–254)	<.001
Total amount of clinical visits			
Local hospital / practitioner	—	357 (72.0)	
University hospital (MUI)	205 (100)	143 (28.0)	
Travel conditions to local site			
Distance, median (IQR), km	25 (23–26)	33 (4–45)	.880
Time, median (IQR), min	28 (28–42)	36 (10–56)	.880
Duration of maintenance, weeks			
Per patient, mean (SD)	37.6 (14.2)	34.0 (15.8)	.704
Total sum	188	578	
At time of data extraction			.962
Therapy completed	2 (40.0)	7 (41.2)	
Therapy ongoing	3 (60.0)	10 (58.8)	

*Note*: Unless indicated otherwise, data are expressed as No. (%) of patients. *p‐*value was calculated using Chi‐Square test for categorical variables and Mann–Whitney *U* test for age as a continuous variable.

Among all participants, nine (40.9%) successfully completed maintenance therapy, while ongoing therapy continued for twelve (59.1%). The mean observation time was similar between the groups (37.6 weeks [SD 14.2] vs. 34.0 weeks [SD 15.8], urban vs. rural, respectively; *p* = .704; Table 1).

Before the implementation of IHCN, rural patients faced significantly higher travel burdens than urban patients, with median two‐way driving distance of 218 (IQR, 154–280) km and median travel time of 162 (IQR, 130–254) minutes per visit at the MUI (*p* < .0001; Table 1).

### 3.2 Reduction of in‐person visits at MUI for rural patients

**FIGURE 1:**
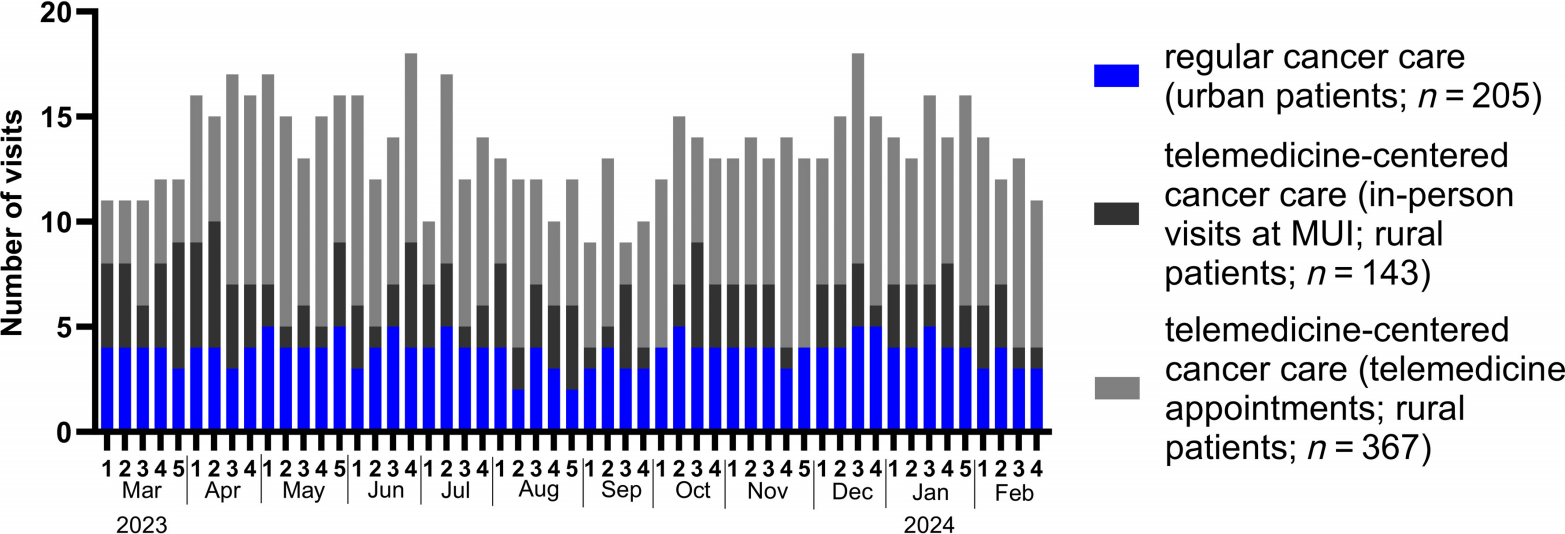
Allocation of routine clinical visits during maintenance for childhood leukemia. Since March 1, 2023, the IHCN has been offered to all eligible rural patients and their families (*n* = 17). Five patients and their families with a one‐way travel distance <30 km (urban) remained in the regular cancer care. Number of weekly visits was allocated to (i) urban patients with regular cancer care (blue) and (ii) rural patients with telemedicine‐centered cancer care separated for in‐person visits at MUI (black) and telemedicine appointments (grey).

As of February 29, 2024, the majority of clinical visits for rural patients (*n* = 367; 72.0%) were conducted via telemedicine, while a small number (*n* = 143; 28.0%) were in‐person visits at the MUI (Figure 1 and Table 1). Urban patients, who received only regular cancer care, had 205 in‐person visits at the MUI (Figure 1 and Table 1). Clinical examinations for rural patients were performed either at the local hospital (10 patients, 58.8%) or by the general practitioner (7 patients, 41.2%) during the morning hours (Table 1), followed by video call (*n* = 318, 86.6%) or conducted by telephone (*n* = 49, 13.4%) later in the day to discuss results with the pediatric oncologists from the MUI.

Results of the physical examinations and laboratory tests were available in 361 cases (98.4%) before the scheduled start of the video consultation, with parents providing results in six cases during the appointments (1.6%).

Following the implementation of IHCN, the median two‐way driving distance for rural patients decreased to 33 (IQR, 4–45) km and 36 (IQR, 10–56) min, which was comparable to that of urban patients (25 [IQR, 23–26] km and 28 [IQR, 28–42] min; *p* = .880; Table 1).

Overall, rural patients saved a total of 70,158 km, 950 h, 12.3 tons of CO_2_, and € 29,467 during the first year (Table S1).

### 3.3 Safety and quality of care

In total, out of all 715 routine clinical visits, 137 (19.2%) revealed new abnormal findings on physical examination, with slight differences between regular cancer care for urban patients (*n* = 46, 22.4%) and telemedicine‐centered cancer care for rural patients (*n* = 91, 17.8%; *p* = .312). These diagnoses were categorized into ICD‐10 chapters, highlighting that most of the abnormal findings were associated with diseases of the respiratory system, particularly acute nasopharyngitis (Table 2, individual ICD codes are listed in Table S2), with no significant variance between both patient cohorts (*χ* ^2^(9) = 10.49, *p* = .312, Cramer‐V = .27).

**TABLE 2:** Identified diagnosis allocated to ICD‐10 chapters.

ICD‐10 chapters	Regular cancer care (urban patients)	Telemedicine‐centered cancer care^a^ (rural patients)
I. Certain infectious and parasitic diseases	6 (13.0)	10 (10.2)
IV. Endocrine, nutritional and metabolic diseases	0	1 (1.0)
VII. Diseases of the eye and adnexa	1 (2.2)	3 (3.1)
VIII. Diseases of the ear and mastoid process	3 (6.5)	7 (7.1)
X. Diseases of the respiratory system	26 (56.5)	42 (42.9)
XI. Diseases of the digestive system	1 (2.2)	15 (15.3)
XII. Diseases of the skin and subcutaneous tissue	2 (4.3)	5 (5.1)
XIII. Diseases of the musculoskeletal system and connective tissue	2 (4.3)	1 (1.0)
XIV. Diseases of the genitourinary system	0	4 (4.1)
XVIII. Symptoms, signs and abnormal clinical and laboratory findings, not elsewhere classified	5 (10.9)	10 (10.2)
Total	46 (100)	98 (100)

*Note*: Data are expressed as No. (%) of diagnosis for regular cancer care (urban patients) and telemedicine‐centered cancer care (rural patients). Chi‐Square test was used to compare both groups and diagnosis allocated to ICD‐10 chapters (*χ* ^2^(9) = 10.49, *p* = .312, Cramer‐V = 0.27).

^a^ Seven telemedicine‐centered appointments were associated with two abnormal findings.

Seven appointments (1.4%) of patients in the telemedicine‐centered cancer care group required an unplanned inpatient stay. Among them, five were due to viral infections (including two with SARS‐CoV‐2), one due to elevated CRP levels combined with fever of unknown origin, and one due to gastroenteritis. Urban patients receiving regular cancer care had a similar frequency of unplanned inpatient stays (*n* = 2; 1.0%; *p* = .219), one due to erysipelas and the other due to elevated CRP levels combined with fever of unknown origin.

Regarding laboratory tests, there was no significant difference in the frequency of leucocyte levels outside the targeted range (1500–3000/μL; 45.7% vs. 47.0%; *p* = .95), neutrophil count below 0.5 G/L (2.6% vs. 5.3%; *p* = .13) and elevated transaminases (16.5% vs. 17.0%; *p* = .97) between regular cancer care and telemedicine‐centered cancer care, respectively. Only urban patients had significantly more often elevated CRP levels than rural patients (23.6% vs. 8.0%, respectively; *p* < .001).

The weekly adjustment of maintenance therapy (including 6‐mercaptopurine, methotrexate, and thioguanine) based on blood count showed comparable rates of dose adaptation in both patient groups (45.9% vs. 44.5%, urban vs. rural, respectively; *p* = .85), as well as similar rates of therapy interruption (8.1% vs. 7.3%, urban vs. rural, respectively; *p* = .85).

### 3.4 Satisfaction and quality of life

**FIGURE 2:**
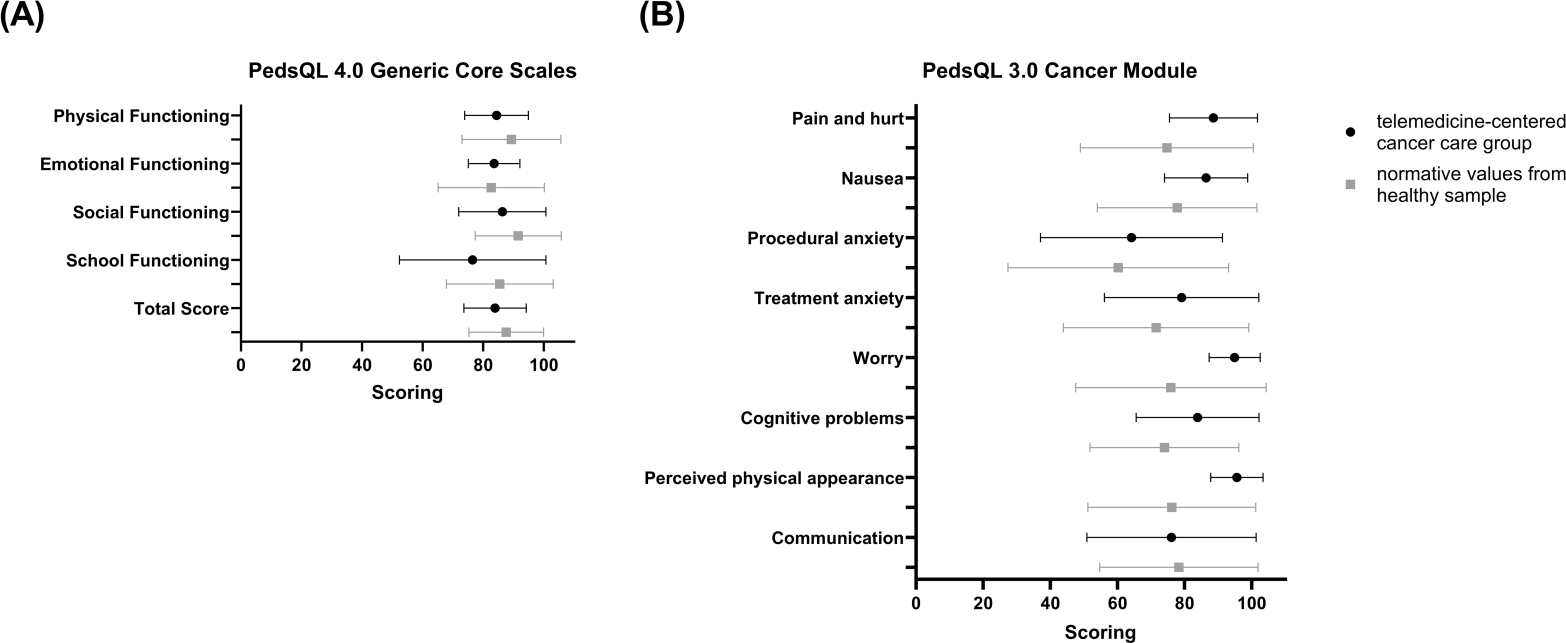
Quality of life assessment. Twelve parents (70.1%) completed the proxy version of the Pediatric Quality‐of‐Life Inventory (PedsQL) 4.0 Generic Core Scales (A) and PedsQL 3.0 Cancer Module (B). Items of the questionnaires were allocated to corresponding dimensions. Data from this cohort (black) and normative values from healthy sample^21^ (grey) are shown as mean with standard deviation.

During maintenance therapy, proxies of rural patients were approached to complete the PedsQL 4.0 Generic Core Scales and the PedsQL 3.0 Cancer Module, providing insights about the HRQOL of their child. Twelve parents (70.1%) responded to both questionnaires, and all subscales indicated no impairment of the quality of life (defined as worse than one SD below normative means, Figure 2A,B).

**FIGURE 3:**
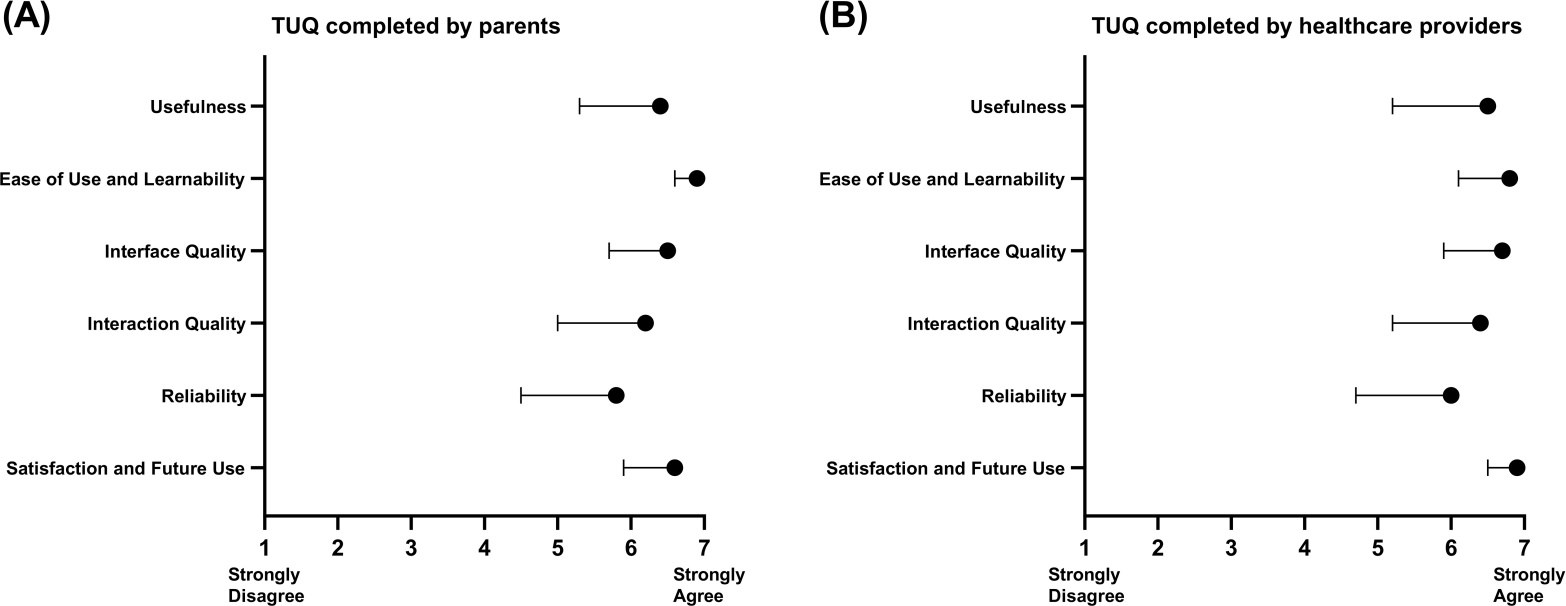
Usability evaluation of IHCN according to the Telehealth Usability Questionnaire (TUQ). Parents of all 17 rural patients (A) and 11 health care providers (B) completed the survey anonymously. The TUQ consists of a 21‐item questionnaire assigned to six subscales as indicated by the respective grading “usefulness,” “ease of use and learnability,” “interface quality,” “interaction quality,” “reliability,” and “satisfaction and future use.” Each question was scored on a 7‐point Likert scale ranging from 1 (strongly disagree) to 7 (strongly agree). Data are shown as mean with standard deviation.

Additionally, all 17 rural participants completed the survey on user satisfaction (TUQ), revealing very high scores for the use of IHCN, along with high confidence and willingness to use telemedicine again (Figure 3A). Practitioner satisfaction mirrored that of participants (Figure 3B). In detail, mean TUQ scores were 6.4 (SD, 0.4) for rural families and 6.5 (SD, 0.6) for health care providers, easily exceeding the score of 5 that is considered useful. The subscales with the highest score were for both user groups, “ease of use and learnability” (6.9 [SD, 0.3] for rural families and 6.8 [SD, 0.7] for health care provider) and “satisfaction and future use” (6.6 [SD, 0.7] for rural families and 6.9 [SD, 0.4] for health care provider). Detailed responses to each individual question are provided in Figures S1 and S2.

## 4 DISCUSSION

Delivering specialized care often involves centralizing health care services into tertiary centers to ensure adequate quality. However, for patients residing far from these centers, this means they face long travel distances to receive care. Concerns about the sustainability of this centralized health care model are mounting, especially considering the increasing costs of transportation and the effects of climate change.^11^, ^24^, ^25^ The COVID‐19 pandemic gave rise to the adoption of innovative care delivery methods, like telemedicine services, which have garnered widespread praise for their advantages.^11^, ^14^, ^26^ Despite this, telemedicine is still not widely utilized in pediatric care settings.

We prioritized implementing the IHCN for acute leukemia patients due to the prolonged treatment duration, necessitating weekly blood count measurements during maintenance phase.^27^ In the first year of the IHCN, over 70% of all in‐person visits at the MUI were replaced. As a result, patients and families traveled a total of 11,805 km instead of 81,964 km, and emissions were reduced from 14.3 tons to just 2 tons of CO_2_ (Table S1).

Implementing telemedicine in pediatric cancer poses unique challenges due to the specialized nature of pediatric care, such as the necessity for tailored treatments and the crucial aspect of maintain trust and rapport with young patients and their families. It is therefore mandatory that our new approach is evaluated by both user groups: the families and the health care provider. For this purpose, we used one of the most frequently used questionnaires for the evaluation of telemedicine applications.^23^, ^28^ The high satisfaction with our IHCN is evident from outstanding mean scores given by rural families and health care providers (6.4 [SD, 0.4] vs. 6.5 [SD, 0.6], respectively) and is comparable with other successfully rated telemedicine services.^29^, ^30^ In addition, various high ratings on single survey questions indicated the effectiveness and convenience of our telemedicine approach (“Telemedicine saves me travel time,” “I would use telemedicine services again,” and “Overall, I am satisfied with the video consultation”; Figures S1 and S2). Notably, the commitment to the IHCN is underscored by the fact that none of the patients or families opted to discontinue the service or return to standard of care visits. Moreover, ensuring equitable access to telemedicine services, including physical examinations and laboratory tests, was prioritized regardless of the socioeconomic factors or geographical location, with some of the families even extending the approach by arranging examinations during their vacations with local physicians. In addition to providing clinical care, regular assessments of the patient's quality of life using the app ePROtect indicated that the scores consistently fall within the normative range observed in healthy peers.^21^ We conclude, that psychosocial and functional outcomes among pediatric leukemia patients using telemedicine‐supported care are favorable.

Our study aimed to assess the financial burden of implementing a two‐pronged approach involving examinations with local general practitioners and the telemedicine consultations with a tertiary hospital for pediatric cancer. We acknowledge the complexity of conducting a comprehensive calculation for such a model. However, our findings demonstrate a significant reduction in transportation costs, thereby highlighting one tangible benefit of this approach. Furthermore, we recognize saving of the additional costs incurred by families for travel to the hospital, such as lost wages due to missed work and childcare expenses. While questions may arise regarding the overall cost‐effectiveness, it is essential to consider the utilization of existing local infrastructures and potential savings, particularly in the case of expensive and complex health care structures like university hospitals.

One frequently debated aspect pertains to data protection and liability concerns associated with telemedicine. Particularly in Austria, notable advantages exist due to the presence of a nationwide electronic health record system, which allows patients, practitioners and hospitals to access the same medical records via dedicated applications or clinical information portals. Within our IHCN, stringent security measures are implemented, including patient authentication via PIN and data storage with the hospital's intranet. Medical liability is assured through adherence to established standards of care during clinical visits, laboratory procedures, and consultations. A major concern as a potential source for mistakes or variability might occur due to different collection sites and laboratory standards. However, in our study, the results of complete blood count measures consistently demonstrated reliability and accuracy, with abnormal findings, such as elevated CRP, confirmed when necessary at MUI. Out of 367 cases, only one patient (0.3%) exhibited an incorrect measurement of low hemoglobin during follow‐up. Moreover, unplanned inpatient stays were required in only seven cases (1.4%) within telemedicine‐centered cancer care group, aligning with a retrospective analysis of the Hospital for Sick Children in Toronto, Canada, which identified only six out of 240 maintenance visits (2.5%) as “in‐person visit essential.”^31^ Our real‐world findings underscore the feasibility of implementing alternative care delivery models, beyond traditional in‐person visits at tertiary centers.

## 5 LIMITATIONS

The findings of this study are limited by its single‐center design, the relatively short duration of 12 months, and the homogeneous patient population, which restrict the generalizability of the results. In addition, we acknowledge that travel savings could only be estimated using Google Maps and that a cost‐effectiveness assessment from the health care provider's perspective is missing. Due to the design of our pilot study, satisfaction with the IHCN was quantified without a comparator, and for the assessment of quality of life, normative values from the literature were used. While it was anticipated, that the study would involve a small number of patients due the rarity of disease, the stated objective was to introduce an IHCN for maintenance therapy. Future research should aim to include a more diverse and larger sample size through multi‐center studies to validate these findings and broaden their applicability.

## 6 CONCLUSIONS

This prospective pilot study shows that a telemedicine‐based IHCN can be implemented in the routine clinical care of pediatric leukemia patients and ensures quality of care, cost savings, safety, and satisfaction on the part of family and clinicians. With the findings presented here, there's now potential to scale up, both in a multi‐center context and across more prevalent adult cancers. But first, important steps still need to be taken, including addressing regulatory issues (e.g., liability, responsibilities) and reaching agreements with health insurance companies (e.g., reimbursement).

## AUTHOR CONTRIBUTIONS

**Andreas Meryk:** Conceptualization; investigation; formal analysis; data curation; writing – review and editing; writing – original draft; visualization. **Christina Salvador:** Investigation; writing – review and editing. **Gabriele Kropshofer:** Investigation; writing – review and editing. **Benjamin Hetzer:** Investigation; writing – review and editing. **Gerhard Rumpold:** Conceptualization; writing – review and editing. **Alexandra Haid:** Investigation; writing – review and editing. **Verena Schneeberger‐Carta:** Investigation; writing – review and editing. **Bernhard Holzner:** Conceptualization; writing – review and editing. **Roman Crazzolara:** Conceptualization; formal analysis; data curation; funding acquisition; writing – original draft; writing – review and editing; project administration.

## CONFLICT OF INTEREST STATEMENT

Bernhard Holzner and Gerhard Rumpold hold intellectual property rights to the software tool CHES. The remaining authors have no conflicts of interests to disclose.

## ETHICS STATEMENT

The ethics committee (EC) at the MUI approved this study (EC number: 1055/2020); written informed consent was obtained from all children 5 years and older and from the caregivers of all children. The study followed the Strengthening the Reporting of Observational Studies in Epidemiology (STROBE) reporting guideline.

## Supporting information


**Data S1.** Supporting Information.

## Data Availability

The datasets generated during and/or analyzed during the current study are available from the corresponding author on reasonable request.
