# Supplementary material for: Pioneering sustainable treatment delivery in childhood leukemia through synchronous telemedicine—A pilot study
Source: Int J Cancer. 2024 Nov 9;156(6):1247–55. doi: 10.1002/ijc.35253 (PMC11737001; doi:10.1002/ijc.35253)
Supplement: Supplementary file 1 — Data S1. Supporting Information. [file IJC-156-1247-s001.pdf]

PIONEERING SUSTAINABLE TREATMENT DELIVERY IN CHILDHOOD LEUKEMIA THROUGH SYNCHRONOUS TELEMEDICINE – A PILOT STUDY

Authors:

Andreas Meryk, Christina Salvador, Gabriele Kropshofer, Benjamin Hetzer, Gerhard Rumpold, Alexandra Haid, Verena Schneeberger-Carta, Bernhard Holzner, Roman Crazzolara

Table of Contents:

- Supplemental Table 1. Calculation of savings by implementation of the telemedicine-based IHCN
- Supplemental Table 2. Identified diagnosis according to ICD-10
- Supplemental Figure 1: Usability evaluation of IHCN according to the Telehealth Usability Questionnaire (TUQ)
- Supplemental Figure 2: Usability evaluation of IHCN according to the Telehealth Usability Questionnaire (TUQ

Supplemental Table 1. Calculation of savings by implementation of the telemedicine-based IHCN

	Before the IHCN (all appointments at MUI)					Implementation of the IHCN (hybrid model of in-person visits at MUI and telemedicine-based IHCN)						Savings			
	Total amount of clinical visits	Distance (km)	Time (min)	CO_2 (kg)	Cost (€)	Amount of telemedicine appointments	Amount of in-person visits at MUI	Distance (km)	Time (min)	CO_2 (kg)	Cost (€)	Distance (km)	Time (min)	CO_2 (kg)	Cost (€)
Patient 1	29	4,466	4,350	782	1,876	21	8	2,261	2,292	396	950	2,205	2,058	386	926
Patient 2	45	12,600	11,880	2,205	5,292	33	12	4,812	5,412	842	2,021	7,788	6,468	1,363	3,271
Patient 3	16	4,096	4,128	717	1,720	12	4	1,600	1,824	280	672	2,496	2,304	437	1,048
Patient 4	35	10,710	8,890	1,874	4,498	33	2	2,460	2,554	431	1,033	8,250	6,336	1,444	3,465
Patient 5	17	2,040	1,938	357	857	14	3	598	706	105	251	1,442	1,232	252	606
Patient 6	42	13,440	13,020	2,352	5,645	31	11	4,543	4,526	795	1,908	8,897	8,494	1,557	3,737
Patient 7	15	4,920	3,840	861	2,067	13	2	864	876	151	363	4,056	2,964	710	1,704
Patient 8	47	11,656	11,092	2,040	4,896	30	17	5,566	5,692	974	2,338	6,090	5,400	1,066	2,558
Patient 9	32	3,776	3,008	661	1,586	24	8	992	896	174	417	2,784	2,112	487	1,169
Patient 10	50	7,000	4,900	1,225	2,940	25	25	3,550	2,550	621	1,491	3,450	2,350	604	1,449
Patient 11	15	3,000	2,430	525	1,260	12	3	1,128	1,158	197	474	1,872	1,272	328	786
Patient 12	63	10,458	8,190	1,830	4,392	48	15	2,682	2,430	469	1,126	7,776	5,760	1,361	3,266
Patient 13	8	1,872	1,456	328	786	7	1	507	518	89	213	1,365	938	239	573
Patient 14	36	10,224	8,496	1,789	4,294	33	3	3,657	2,754	640	1,536	6,567	5,742	1,149	2,758
Patient 15	28	4,088	3,024	715	1,717	17	11	1,640	1,256	287	689	2,448	1,768	428	1,028
Patient 16	10	1,610	1,400	282	676	4	6	968	848	169	406	642	552	112	270
Patient 17	22	4,796	3,344	839	2,014	10	12	2,766	2,104	484	1,161	2,030	1,240	355	853
**Total**	**510**	**110,752**	**95,386**	**19,382**	**46,516**	**367**	**143**	**40,594**	**38,396**	**7,104**	**17,049**	**70,158**	**56,990**	**12,278**	**29,467**

Supplemental Table 2. Identified diagnosis according to ICD-10

**ICD-10**	**regular cancer care**	**telemedicine-centered cancer care***
Acute nasopharyngitis	16 (34.8)	21 (21.4)
Acute upper respiratory infect	7 (15.2)	16 (16.3)
Otitis media	3 (6.5)	7 (7.1)
Herpesviral infections	4 (8.7)	7 (7.1)
Other specified fever	3 (6.5)	6 (6.1)
Gastritis	0	4 (4.1)
Gastroenteritis	1 (2.2)	4 (4.1)
Urinary tract infection	0	4 (4.1)
Atopic dermatitis	2 (4.3)	4 (4.1)
Conjunctivitis	1 (2.2)	3 (3.1)
Constipation	0	3 (3.1)
Nausea and vomiting	1 (2.2)	2 (2.0)
Acute abdomen	1 (2.2)	2 (2.0)
Orale aphthen	0	2 (2.0)
Acute laryngitis	0	3 (3.1)
Haemarthrosis	0	1 (1.0)
Toothache	0	1 (1.0)
Viral warts	0	1 (1.0)
Varicella without complication	0	1 (1.0)
Acute bronchitis	3 (6.5)	1 (1.0)
Streptococcal tonsillitis	0	1 (1.0)
Diarrhea	0	2 (2.0)
Urticaria	0	1 (1.0)
Hypokalemia	0	1 (1.0)
Osteonecrosis	1 (2.2)	0
Erysipelas	1 (2.2)	0
Cytomegalovirus hepatitis	1 (2.2)	0
Transient synovitis of hip	1 (2.2)	0
**Total**	46 (100)	98 (100)

Data are expressed as No. (%) of diagnosis for regular cancer care (urban patients) and telemedicine-centered cancer care (rural patients). Chi-Square test was used to compare both groups and diagnosis according to ICD-10 ($\chi^2(29) = 26.42$, P=.603, Cramer-V = .428)

* six telemedicine-centered appointments were associated with two abnormal findings

Supplemental Figure 1

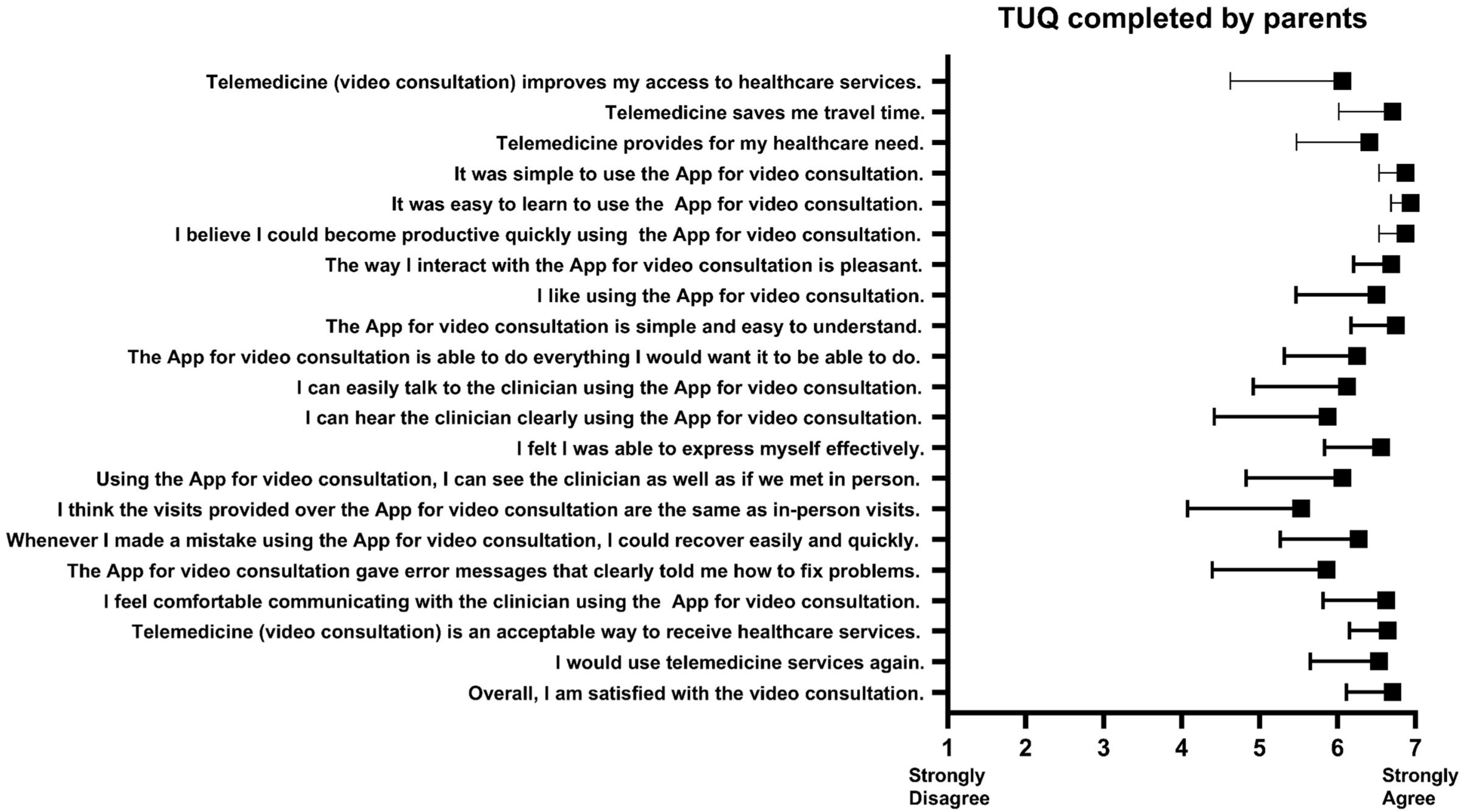

Supplemental Figure 1: Usability evaluation of IHCN according to the Telehealth Usability Questionnaire (TUQ). Parents of all 17 rural patients completed the survey anonymously. The TUQ consists of a 21-item questionnaire and response to each question is shown. Each question was scored on a 7-point Likert scale ranging from 1 (strongly disagree) to 7 (strongly agree). Data are shown as mean with standard deviation.

Supplemental Figure 2

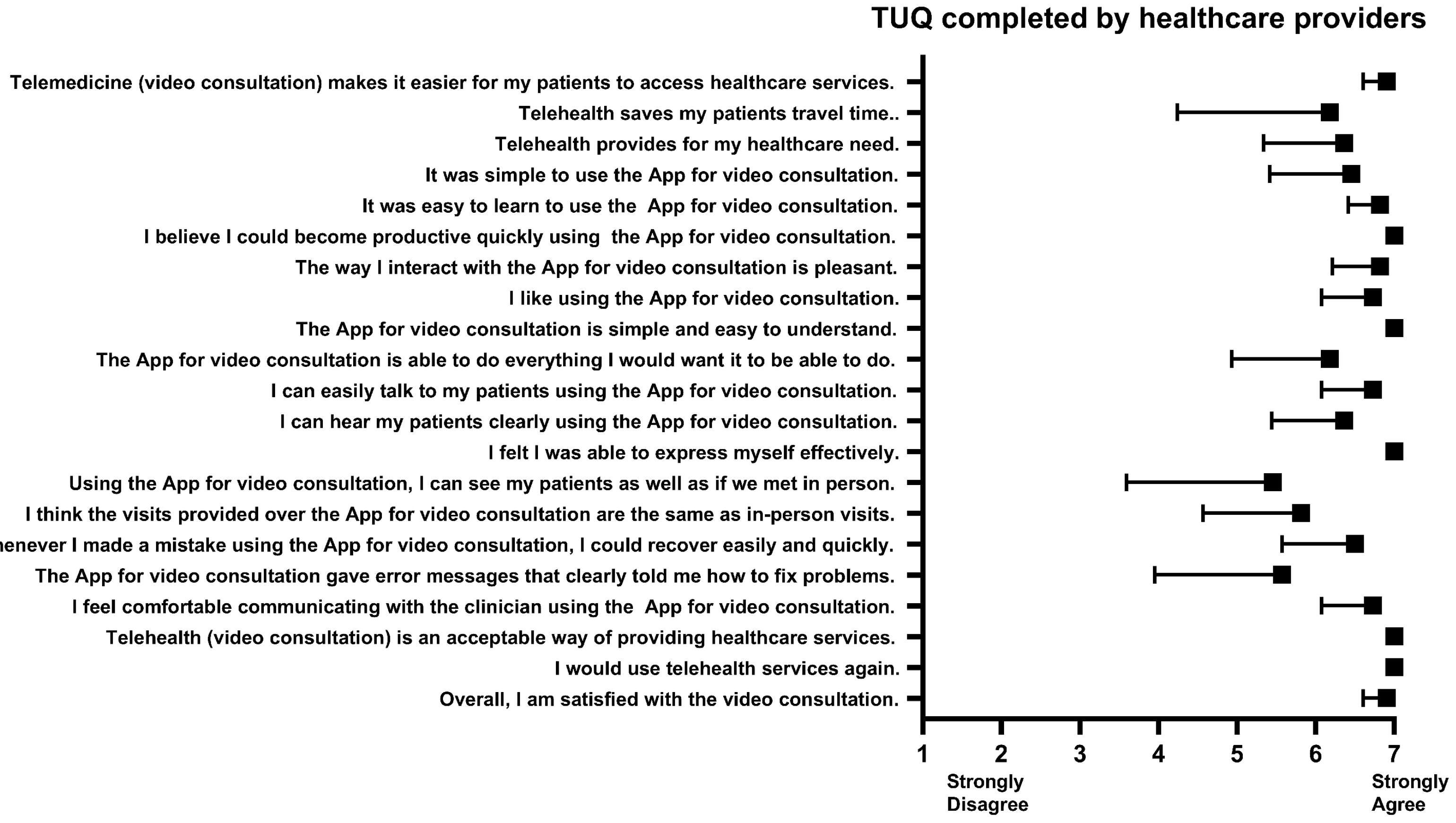

Supplemental Figure 2: Usability evaluation of IHCN according to the Telehealth Usability Questionnaire (TUQ). Eleven healthcare provider completed the survey anonymously. The TUQ consists of a 21-item questionnaire and response to each question is shown. Each question was scored on a 7-point Likert scale ranging from 1 (strongly disagree) to 7 (strongly agree). Data are shown as mean with standard deviation.
